# Point-of-care lung ultrasound for the detection of pulmonary manifestations of malaria and sepsis: An observational study

**DOI:** 10.1371/journal.pone.0204832

**Published:** 2018-12-12

**Authors:** Stije J. Leopold, Aniruddha Ghose, Katherine A. Plewes, Subash Mazumder, Luigi Pisani, Hugh W. F. Kingston, Sujat Paul, Anupam Barua, M. Abdus Sattar, Michaëla A. M. Huson, Andrew P. Walden, Patricia C. Henwood, Elisabeth D. Riviello, Marcus J. Schultz, Nicholas P. J. Day, Asok Kumar Dutta, Nicholas J. White, Arjen M. Dondorp

**Affiliations:** 1 Mahidol-Oxford Tropical Medicine Research Unit, Faculty of Tropical Medicine, Mahidol University, Bangkok, Thailand; 2 Centre for Tropical Medicine and Global Health, Nuffield Department of Medicine, University of Oxford, Oxford, United Kingdom; 3 Department of Internal Medicine, Chittagong Medical College Hospital, Chittagong, Bangladesh; 4 Department of Radiology, Chittagong Medical College Hospital, Chittagong, Bangladesh; 5 Department of Intensive Care, Academic Medical Center, University of Amsterdam, Amsterdam, The Netherlands; 6 Department of Intensive Care, Royal Berkshire Hospital, Reading, United Kingdom; 7 Department of Emergency Medicine, Brigham and Woman’s Hospital, Boston, Massachusetts, United States of America; 8 Division of Pulmonary, Critical Care and Sleep Medicine, Beth Israel Deaconess Medical Center and Harvard Medical School, Boston, Massachusetts, United States of America; Universidade de Sao Paulo Instituto de Ciencias Biomedicas, BRAZIL

## Abstract

**Introduction:**

Patients with severe malaria or sepsis are at risk of developing life-threatening acute respiratory distress syndrome (ARDS). The objective of this study was to evaluate point-of-care lung ultrasound as a novel tool to determine the prevalence and early signs of ARDS in a resource-limited setting among patients with severe malaria or sepsis.

**Materials and methods:**

Serial point-of-care lung ultrasound studies were performed on four consecutive days in a planned sub study of an observational cohort of patients with malaria or sepsis in Bangladesh. We quantified aeration patterns across 12 lung regions. ARDS was defined according to the Kigali Modification of the Berlin Definition.

**Results:**

Of 102 patients enrolled, 71 had sepsis and 31 had malaria. Normal lung ultrasound findings were observed in 44 patients on enrolment and associated with 7% case fatality. ARDS was detected in 10 patients on enrolment and associated with 90% case fatality. All patients with ARDS had sepsis, 4 had underlying pneumonia. Two patients developing ARDS during hospitalisation already had reduced aeration patterns on enrolment. The SpO_2_/FiO_2_ ratio combined with the number of regions with reduced aeration was a strong prognosticator for mortality in patients with sepsis (AUROC 91.5% (95% Confidence Interval: 84.6%-98.4%)).

**Conclusions:**

This study demonstrates the potential usefulness of point-of-care lung ultrasound to detect lung abnormalities in patients with malaria or sepsis in a resource-constrained hospital setting. LUS was highly feasible and allowed to accurately identify patients at risk of death in a resource limited setting.

## Introduction

Respiratory distress is common in patients with malaria or sepsis. A major underlying life-threatening cause of respiratory distress includes acute respiratory distress syndrome (ARDS) [1,2]. During ARDS, fluid accumulates into the lung interstitium and alveolar space resulting in reduced oxygen diffusion [3]. Early bedside diagnosis of life-threatening ARDS may guide therapy, which could eventually improve outcomes.

Point-of-care lung ultrasound (LUS) is a non-invasive and sensitive tool for real-time imaging of lung aeration. Normal lung aeration can be assessed by determining the presence of A-lines with normal lung sliding. Reduction of lung aeration can be observed if so-called B-patterns are present: multiple long vertical ultrasound artefacts caused by lung congestion [4–6]. In addition, lung consolidations are detectable by their characteristic tissue pattern (hepatisation of lung tissue), in some cases combined with a static or dynamic air bronchogram [7]. Combining these aeration patterns in composite outcome measures can provide information about diffuse interstitial fluid filling and life-threatening ARDS [8–10]. In previous studies, lung ultrasound has shown to outperform chest radiography in the detection of lung oedema [11]. Recently, a modification of the international consensus definition of ARDS (the Berlin Definition) has been proposed to facilitate a diagnosis of ARDS based on lung ultrasound and SpO_2_/FiO_2_ (SF) ratios in resource-limited settings [12,13]. A recent observational study in an intensive care unit in the Netherlands found high diagnostic agreement between the Berlin Definition and the new Kigali Modification [14].

We aimed to describe and quantify LUS abnormalities to determine the presence of potentially life-threatening ARDS in patients with malaria or sepsis admitted to a large tertiary referral hospital in Bangladesh. We hypothesised that LUS might detect early pulmonary abnormalities in these patients, even before hypoxemia or respiratory distress is present.

## Methods

### Study design and setting

We conducted a prospective observational cohort study of admitted patients >12 years old with malaria or other sepsis syndromes. Consecutive patients were enrolled over a 10-week study period. All patients were enrolled in the general medical wards of Chittagong Medical College, a tertiary government hospital in Chittagong, Bangladesh, a city of approximately 5 million people. At the time of the study, the hospital had three medical wards with 66 beds each; on an average week day, the bed occupancy rate reaches over 150%. Oxygen was available on all of the study wards and haemodialysis was available in a separate renal unit operating 8 hemofiltration machines. The hospital has an intensive care unit with 12 beds of which 7 are equipped with mechanical ventilators.

### Ethics

This was a component of a larger observational study approved by the Chittagong Medical College Ethics Committee as well as the Oxford Tropical Medicine Research Ethics Committee (OxTREC) (ClinicalTrials.gov NCT02451904). All participants provided written informed consent prior to enrolment into the study. Individual written informed consent was obtained from the patient or the patients’ relative in those unable to give consent. In patients under 18 years of age, informed consent was also obtained from the attending parents or guardians. Research physicians received training according to the Guidelines for Good Clinical Practice [15].

### Inclusion and exclusion criteria

#### Severe malaria

Modified World Health Organisation criteria for severe falciparum malaria were used [16]. Patients >12 years old were eligible until 24 hours after start of antimalarial treatment with either parenteral artemisinin or quinine. Severe malaria was defined as any asexual stage *Plasmodium* parasitaemia detected by microscopy or a positive rapid diagnostic test in combination with one or more of: 1) GCS <11; 2) Haematocrit < 20% with parasite count > 100,000/ mm; 3) Jaundice with bilirubin > 40 μmol/L with parasite count > 100,000/ mm; 4) Serum creatinine > 265 μmol/L; 5) Hypoglycaemia with venous glucose < 2.2 mmol/L; 6) Systolic blood pressure < 80 mm Hg with cool extremities; 7) Peripheral asexual stage parasitaemia > 10%; 8) Peripheral venous lactate > 4 mmol/L, 9) Peripheral venous bicarbonate < 15 mmol/L; 10) Respiratory insufficiency.

#### Uncomplicated malaria

Uncomplicated Malaria required the presence of *Plasmodium* asexual stages on a peripheral blood-slide or a positive rapid diagnostic test, in the absence of any of the above severity criteria.

#### Sepsis

Sepsis syndromes other than malaria were defined by previously described criteria [17]. We used the SIRS criteria to define sepsis because of the practical use in resource-constrained settings where arterial blood gases may not be available. Patients >12 years of age were eligible if there was a suspected clinical infection in combination with any two of the following: 1) Heart rate >90 beats per minute; 2) Respiratory rate >20 breaths per minute, a PaCO_2_ of <32 mm Hg, or the use of mechanical ventilation for an acute respiratory process; 3) Tympanic temperature >38°C OR <36°C; 4) either a white-cell count of >12x10^3^/μL or <4x10^3^/μL or a differential count showing 10% immature neutrophil. Malaria had to be excluded by microscopy and a rapid diagnostic test (Paracheck-Pf, Orchid, India) for *falciparum* and *vivax* malaria. Bacterial cultures were not routinely available at the study site and a positive blood culture was not a requirement. Patients were excluded only if they had a known malignancy or a known pre-existing chronic lung disease.

### Screening and data collection

Over a 10-week period during the rainy season in Bangladesh (May-August 2017), we screened consecutive patients with fever for malaria. Provided consent was obtained, patients with a positive malaria test were enrolled and evaluated for severity of disease. Patients negative for malaria were assessed by a study physician for eligibility in the sepsis observational arm.

At baseline, demographic data and a medical history were collected from the patient and/or a family member. Vital signs were recorded and a physical exam was done at baseline and repeated daily until discharge or death; abnormal findings were communicated with the treating ward doctor.

### Lung ultrasound examination

A protocolised LUS examination was performed at baseline and repeated on day 1, 2, and 3 following enrolment. LUS was performed according to a standard operating procedure specifically designed for performing LUS in a resource-limited setting. In brief, the overall procedure aimed to score lung aeration patterns in 12 lung regions in less than 15 minutes. Each hemithorax was divided in 6 lung regions covering the frontal, lateral and dorsolateral areas of the chest. In this study, we used a GE Healthcare Vivid-I portable ultrasound machine (GE Healthcare, Chicago, Illinois) with a pre-programmed lung setting in which automatic image filters were minimised. Lung ultrasound studies were conducted in a parallel plane using a convex (abdominal) 5 MHz probe. Patients were examined in the supine position, unless this was impossible due to severe hypoxemia; these patients were examined in a semi-recumbent position. Ultrasonography data were stored for post-hoc quality assurance.

### Lung ultrasound outcomes and interpretation

#### Lung aeration measures

**Fig 1:**
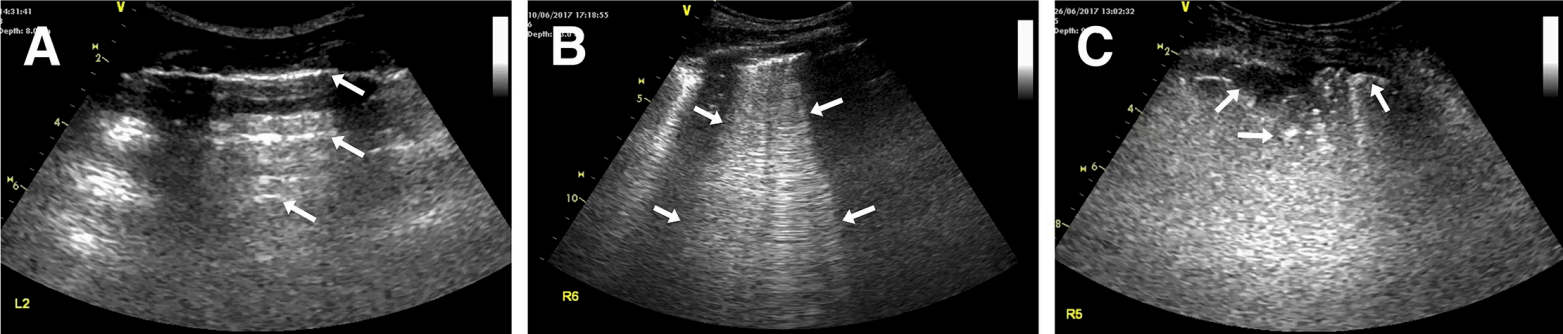
Common lung ultrasound patterns. (A) ‘A-lines’ are reverberation artefacts of the pleura line present in normally aerated lung tissue; they are indicated by the arrows. (B) ‘B-patterns’ are pathological signs of extravascular lung water. Here, multiple coalescent B-lines are shown between the indicator arrows, extending to the lower end of the screen. (C) ‘C-patterns’ are characteristic for pulmonary consolidation suggesting pneumonia. Here, an interruption of the normal pleural line is shown, with multiple echogenic punctiform laesions (as indicated by the arrows) and an underlying air bronchogram.

One trained lung ultrasound examiner scored each of twelve examined lung regions at the bedside. Each score was based on four pre-defined aeration patterns of which the most abnormal score was recorded: 1) Presence of lung sliding with A-lines or maximal two isolated B-lines: indicating normal aeration; 2) Multiple (>2) well-defined B-lines (B1 pattern): moderate loss of lung aeration; 3) Multiple coalescent B-lines (B2 pattern): severe loss of lung aeration; and 4) The presence of a tissue pattern characterised by dynamic air bronchograms without surrounding effusion (C-pattern): lung consolidation [18]. Common ultrasound patterns are shown in Fig 1. We separately recorded effusion(s) and consolidation with effusion.

#### Composite LUS outcomes

LUS bilateral interstitial syndrome was defined as having bilaterally ≥2 areas showing a B-pattern (either B1 or B2 pattern). An interstitial syndrome was considered a sign of increased pulmonary density or reduced aeration, which is usually explained by pulmonary oedema. Less common causes of unilateral interstitial syndrome include regional deflation (atelectasis) or increased tissue mass (tumours, pneumonia).

The Berlin Definition of ARDS included bilateral opacities on chest imaging within one week of a new clinical insult or worsening respiratory symptoms, not fully explained by effusions, lung collapse, nodules, or fluid overload; in combination with impaired oxygenation (SpO_2_/FiO_2_ ≤300) with a minimum positive end-expiratory pressure (PEEP) of ≥5 cm water [12].

The Kigali modification of the Berlin Definition of ARDS [13] for resource-limited settings uses the following criteria for the diagnosis of ARDS: SpO_2_/FiO_2_ ≤315 (with a requirement of SpO_2_ ≤97%) and ≥2 areas on each hemithorax with ‘B-lines’ or consolidation detected by lung ultrasound. In accordance with the Kigali modification, there was no requirement for PEEP. Cardiogenic causes were assessed through focused transthoracic echocardiography (TTE) by measurement of the left ventricular fractional shortening (considered abnormal if ≤25%). Measurement of SpO_2_/FiO_2_ (peripheral oxygen saturation/Fraction of inspired Oxygen) was used for determining an oxygenation cut-off for ARDS and correlates well with PaO_2_/FiO_2_. Oxygen saturation (SpO_2_, S) was measured using a handheld pulse oximeter (H100N, EDAN, China), provided a stable and consistent trace could be established. The fraction of inspired oxygen (FiO_2,_ F) was determined from a read-out of the wall oxygen valve indicator. FiO_2_ was assumed as 21% in each patient breathing room air, and was adjusted by accounting 3 percentage points for each additional litre up to 15L [19].

#### Additional imaging

Chest x-rays (CXR) were recorded on the medical wards using a portable chest X-ray machine in A-P view. CXR were printed and reviewed by an independent senior radiologist (S.M.) who was unaware of the lung ultrasound findings. Focused transthoracic echocardiography (TTE) was done at baseline following lung ultrasonography evaluating left ventricular ejection fraction and inferior vena cava collapsibility. To determine left ventricular fractional shortening (LVFS) the end diastolic diameter (EDD) and end systolic diameter (ESD) were measured in the parasternal long axis view: LVFS(%) = (EDD–ESD / EDD) x 100%; LVFS was considered normal between 25–43%, and severely impaired if below 15% [20]. The subcostal inferior vena cava collapsibility index was measured while the patient was lying supine at 2 to 3 cm from the right atrial border during inspiration and expiration by: ((IVCend-exp—IVC_end-insp_)/ IVC_end-exp_) x 100% [21].

### Statistical analysis

Baseline characteristics and main outcomes were presented as counts and proportions for categorical variables, medians and inter quartile ranges for non-normally distributed variables, and means and standard deviation for normally distributed variables. Analysis was performed on the published datasets: S1 and S2 Datasets.

To determine predictors of death among patients with sepsis, we built a logistic regression model using death as the outcome variable and LUS detected B-patterns and the SF-ratio as predictor variables; Following confirmation of the model assumptions, a logistic regression model was fitted using the ‘*glm*’ package in R. Area under the Receiver Operating Curves Characteristics were calculated with a 95% confidence interval to determine the prognostic value of LUS, SF-ratio, and a combination of both, using the ‘*pROC*’ package in R.

Agreement on lung infiltrates/pulmonary oedema diagnosed by chest radiography and lung ultrasonography was assessed in cases where results were available for both imaging techniques at baseline. Both positive as well as negative agreement was reported, because of the absence of a gold standard. All statistical analyses were done in R statistical software (V3.3.3).

## Results

### Baseline characteristics

**Fig 2:**
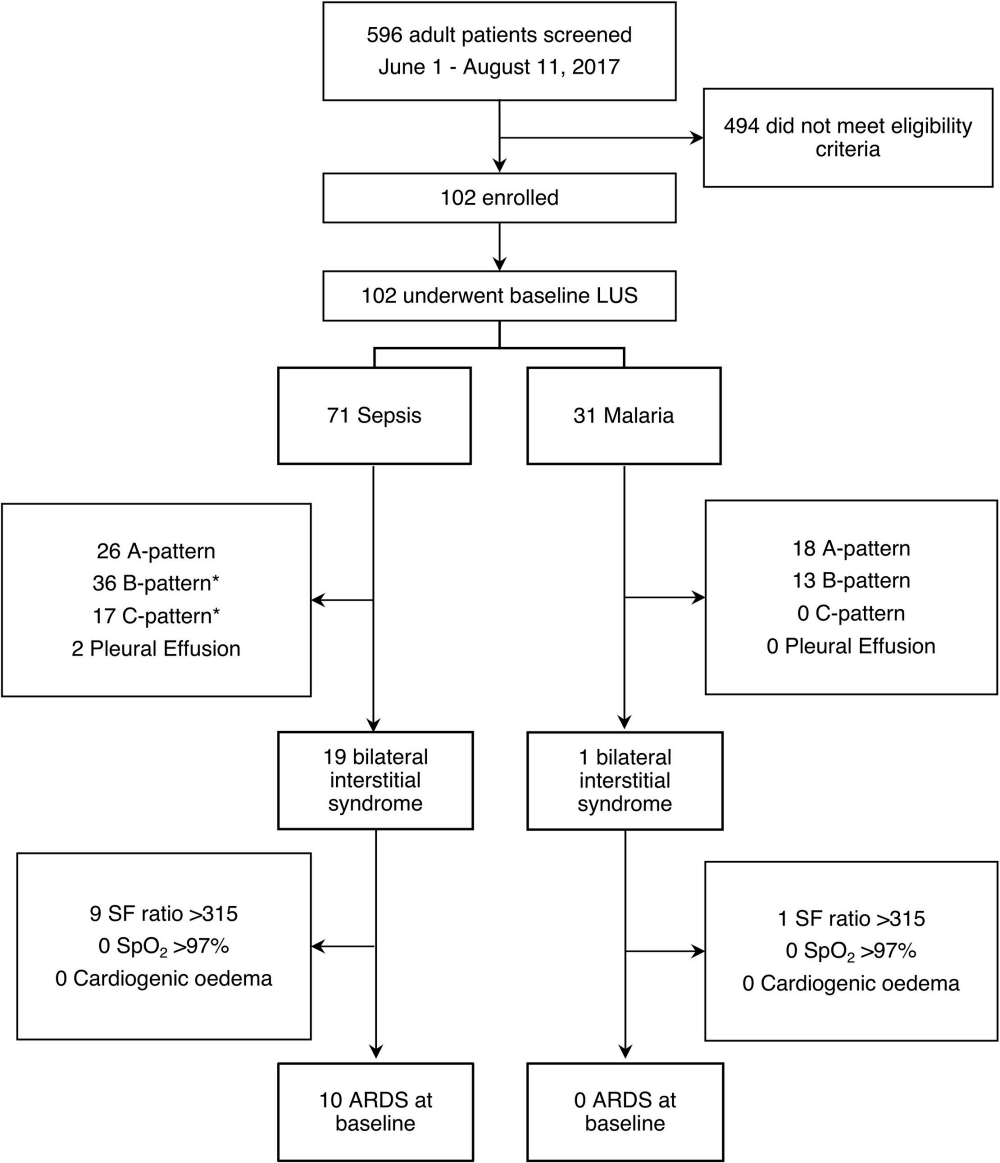
STROBE flow diagram. *Complications can overlap in the same patient.

During a 10-week period between May and August 2017, a total of 596 febrile patients were screened for eligibility (STROBE Flow Diagram, Fig 2). A total of 102 consecutive patients were enrolled into the study. Of these, 31 were diagnosed with malaria (30 *Plasmodium falciparum*, one *Plasmodium vivax*) and 71 patients had other forms of sepsis (Table 1). At baseline, all 102 patients underwent lung ultrasonography, in 90/102 (88%) chest radiography was available in 87/102 (84%) left ventricular fractional shortening could be determined by focussed TTE. All time points combined (Day 0, 1, 2 and 3), a total of 211 serial point-of-care lung ultrasound studies were completed amounting to 2532 scanned lung regions. Of all scanned lung regions 98% were interpretable, the remaining 2% were not interpretable due to technical reasons or because the heart obscured the lung fields. Overall, agreement between CXR and LUS for pulmonary oedema and lung infiltrates/consolidations was 83%. Positive agreement was 56% and negative agreement was 90%. The low positive agreement was due to a higher number of abnormal findings detected through lung ultrasound.

**Table 1:** Patient characteristics and radiological findings at baseline.

	Sepsis	Severe Malaria	Uncomplicated Malaria
***Demographic variables***			
*N*	71	13	18
Male, n(%)	39 (55%)	8 (57%)	14 (78%)
Age (years)	38 (33)	33 (23)	35 (30)
***Disease severity***			
Mortality, n(%)	18 (25%)	4 (31%)	0 (0%)
SF	452 (52)	462 (14)	464 (12)
GCS (3–15)	15 (4)	9 (3)	15 (0)
MABP (mm Hg)	85 (21)	80 (7)	75 (13)
Creatinine (μmol/L)	75 (58)	117 (173)	86 (68)
Base Deficit (mmol/L)	-2 (5)	-6 (7)	-2.5 (3)
Lactate (mmol/L)	1.6 (1)	2.3 (1.6)	1.2 (0.5)
WBC (x10^3^/μL)	11.2 (8.2)	9.2 (5.6)	5.4 (2.8)
Platelets (x10^3^/μL)	202 (166)	31 (48)	55 (45)
***Lung ultrasonography***			
A-pattern (Whole chest normal aeration)	26 (37%)	8 (62%)	10 (56%)
B-pattern (1–4 areas)	19 (27%)	4 (31%)	7 (39%)
B-pattern (5–8 areas) *	10 (14%)	1 (8%)	1 (6%)
B-pattern (9–12 areas) *	7 (10%)	0 (0%)	0 (0%)
C-pattern (≥1 area(s) with consolidation) *	17 (24%)	0 (0%)	0 (0%)
Bilateral Interstitial syndrome *	19 (27%)	0 (0%)	1 (6%)
ARDS (Kigali modification) *	10 (14%)	0 (0%)	0 (0%)
***Echocardiography***			
LVFS (%)	31 (7)	41 (11)	31 (10)
IVC collapsibility (%)	26 (33)	18 (19)	26 (40)
***Chest radiography***			
Any infiltrate	18 (30)	0 (0)	0 (0)

All are medians (Inter Quartile Range, IQR) unless stated otherwise. SF, SpO_2_/FiO_2_ ratio; GCS, Glasgow Coma Scale; MABP, Mean arterial blood pressure; WBC, White Blood Cells. ARDS, Acute Respiratory Distress Syndrome; LVFS, left ventricular fractional shortening. IVC, inferior vena cava.

*Cases may have overlapping LUS abnormalities.

### LUS evidence of pulmonary involvement

Normal lung aeration, defined as an A-pattern observed in all scanning zones with normal lung sliding, was found in 44/102 (43%) patients at baseline. Normal lung aeration was observed in 26/71 (37%) patients with sepsis and 18/31 (58%) patients with malaria (Table 1).

Reduced lung aeration in one or more scanning zones, either moderate (B1-pattern) or severe (B2-pattern), was observed in 49/102 (48%) patients. The majority of patients with reduced lung aeration had between 1 and 4 lung areas showing a B-pattern.

Lung consolidation with dynamic air bronchograms but without surrounding effusion, which is suggestive of pneumonia, was found in 17/102 (17%) patients. None of the patients with malaria had a consolidation at baseline. Pleural effusion was observed in 2/71 (3%) patients with sepsis.

ARDS could not be diagnosed using the conventional Berlin Definition because the requirements for positive end-expiratory pressure criteria were not met in this setting since patients were managed outside of the ICU. ARDS according to the Kigali Modification of the Berlin Definition, using CXR as the only imaging modality, was found in 4/102 (4%) patients. Using LUS we identified 10/102 (10%) patients with ARDS at baseline. Combining LUS with CXR did not yield any additional identification of ARDS. All patients with ARDS had sepsis.

### Longitudinal LUS findings

The majority of LUS abnormalities were observed on enrolment. In the three days following enrolment, 34 out of the initial 102 (33%) patients developed new or worsening LUS abnormalities, details are presented in Table 2. An increasing number of areas containing a B-pattern was the most common finding observed in 24/102 (24%) patients. During in-hospital stay, 8 out of the initial 102 (8%) patients developed a new area with a C-pattern. Two patients with sepsis developed ARDS. Both of the patients who developed ARDS during in-hospital stay showed already a bilateral interstitial syndrome assessed by LUS, yet without severe hypoxia (SF ≤315), which developed later during admission.

**Table 2:** New LUS findings observed during in-hospital stay.

	*Day 0*	*Day 1*	*Day 2*	*Day 3*
**Sepsis (*N*)**	(71)	(51)	(39)	(22)
B-pattern (1–4 areas)	19	6	3	2
B-pattern (5–8 areas)	10	3	1	0
B-pattern (9–12 areas)	7	0	0	1
C-pattern	17	3	2	0
ARDS	10	2	0	0
**Severe Malaria (*N*)**	(13)	(9)	(8)	(6)
B-pattern (1–4 areas)	4	0	0	1
B-pattern (5–8 areas)	1	0	0	1
B-pattern (9–12 areas)	0	0	1	0
C-pattern	0	0	0	0
ARDS	0	0	0	0
**Uncomplicated Malaria (*N*)**	(18)	(13)	(11)	(8)
B-pattern (1–4 areas)	7	4	0	0
B-pattern (5–8 areas)	1	0	0	1
B-pattern (9–12 areas)	0	0	0	0
C-pattern	0	2	1	0
ARDS	0	0	0	0

ARDS: Acute respiratory distress syndrome.

### LUS predictors of fatal outcome

**Fig 3:**
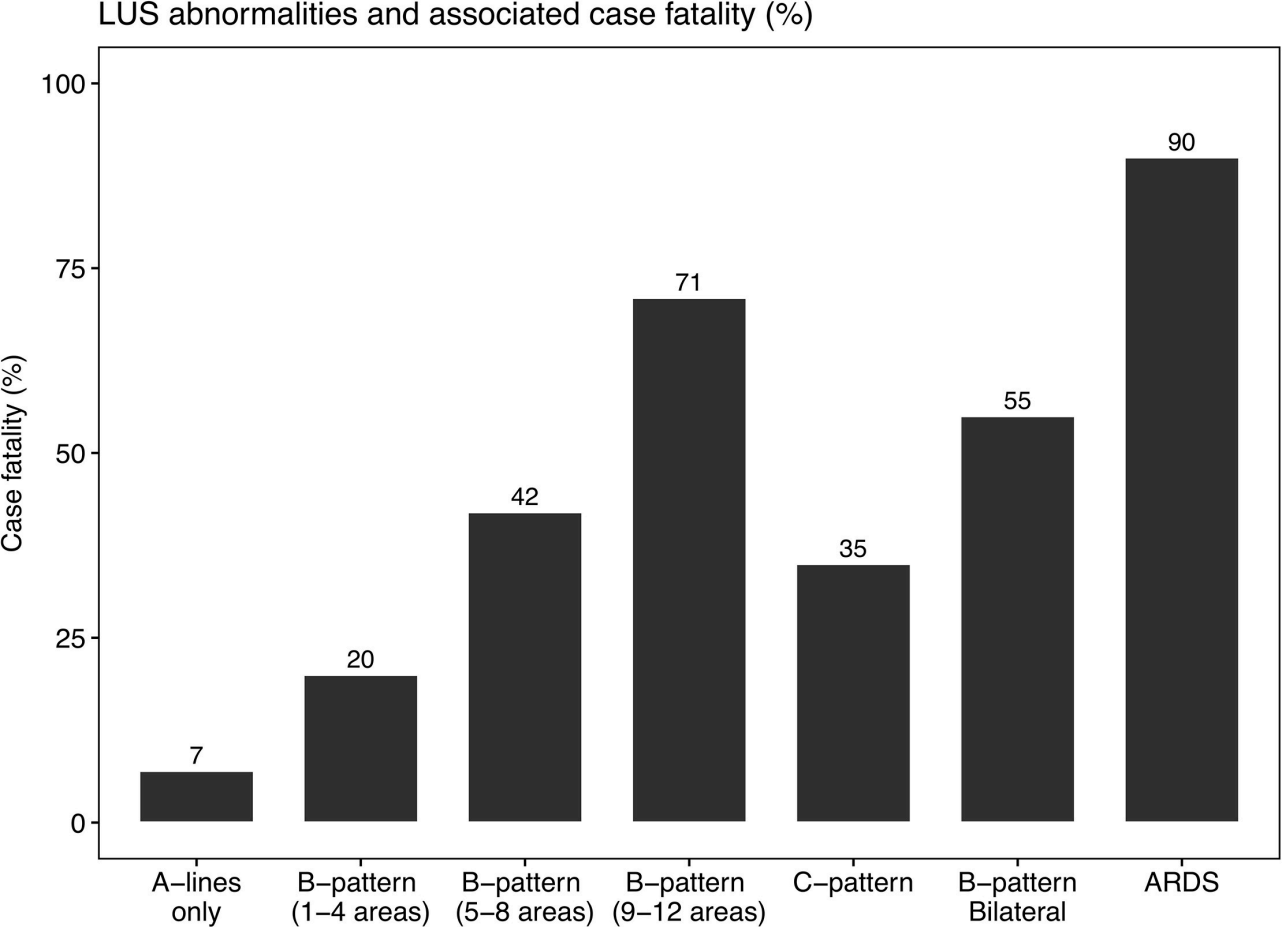
Lung ultrasound observations and associated case fatality rates in patients with sepsis and severe malaria (n = 102) in Bangladesh.

Overall case fatality was 25% in sepsis and 31% in severe malaria. We observed different case fatality rates between patients who had fully normal lung ultrasound exam on enrolment (7%) and patients who had bilateral lung interstitial syndrome (55%) or ARDS (90%) (Fig 3). Of all patients who died, 86% had baseline LUS abnormalities. A higher number of LUS abnormalities was associated with a higher risk of in-hospital death (Fig 3).

**Fig 4:**
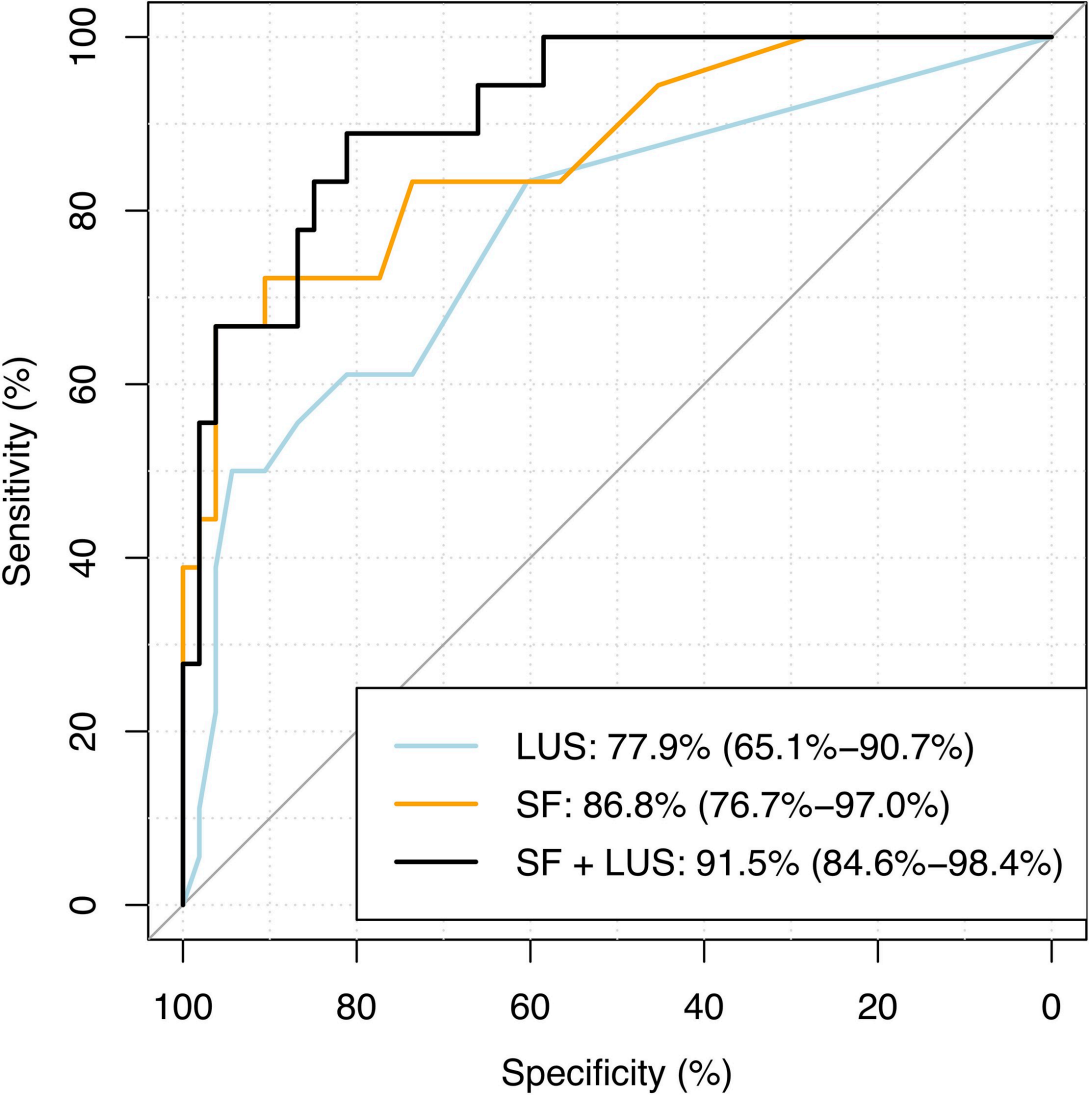
AUROCC of LUS findings, SF ratios, and their combined ability to predict fatal outcome in patients with sepsis (n = 71) in Bangladesh. Area Under the Receiver Operating Characteristics Curve (AUROCC) of 1) Lung ultrasound (LUS) quantification of the number of lung regions (0 to 12) with a B-pattern (B1 or B2); 2) SpO_*2*_/FiO_*2*_ ratios (SF); and 3) SF and LUS combined.

Among patients with sepsis (n = 71), the number of lung areas with a B-pattern on enrolment was predictive of death during in-hospital stay, as shown in Table 3. Combining LUS and SF ratios predicted death with an area under the receiver operating curve (AUROC) of 91% (CI 85%-98%) (Fig 4). B-patterns were most commonly observed in the lateral and posterior lung zones (S1 Fig).

**Table 3:** Logistic regression model to predict in-hospital mortality in patients with sepsis (n = 71) in Bangladesh.

	Univariate Models			Multivariate Model			
Variable	Estimate	Std. Error	P	Estimate	Std. Error	P	
**LUS**	0.286	0.083	0.0006	0.194	0.100	0.052	HL: 6.04, p = 0.64
**SF ratios**	-0.017	0.004	0.00001	-0.016	0.004	0.0001	

LUS: the number of lung areas with a B1 or B2 pattern. SF ratios: SpO_2_/FiO_2_ ratio. HL: Hosmer-Lemeshow goodness of fit test.

The case fatality rate of ARDS found on enrolment was 9/10. Unfortunately, none of the patients with ARDS could be admitted to ICU, because of limited bed capacity. The location of areas of B-pattern was similar between patients with or without ARDS (S2 Fig).

## Discussion

This study demonstrates the potential large benefit of point-of-care LUS in early detection of pulmonary manifestations of malaria and sepsis. We developed a setting-adapted practical standard operating procedure using a 12-region approach. We observed that a normal lung ultrasound assessment on enrolment was associated with low case fatality, whereas an increase in the number of LUS abnormalities was closely associated with higher case fatality. Notably, mortality in ARDS was very high (90%) in the absence of access to positive pressure mechanical ventilation because of limited access to ICU. Most patients with ARDS presented with the condition on enrolment. Those patients developing ARDS later during admission showed on enrolment early signs of bilateral interstitial syndrome prior to the development of severe hypoxemia. The number of chest zones containing a B-pattern appeared a strong predictor of death in patients with sepsis.

This is the first study to report pulmonary manifestations of malaria and sepsis using point-of-care lung ultrasound. These findings highlight the difficulties of diagnosing ARDS in a resource-constrained hospital by conventional criteria and show the potential for adjusted LUS based ARDS criteria to be used for triage of patients at high risk. Early LUS findings of reduced lung aeration may suggest an increased risk of developing ARDS requiring a change in treatment.

We considered a sonographic bilateral diffuse interstitial syndrome, not due to cardiogenic oedema (as assessed through echocardiography), to be primarily resulting from interstitial and alveolar oedema. The pathophysiology of non-cardiogenic pulmonary oedema in severe malaria is different from that in sepsis. In sepsis, ARDS is thought to occur following neutrophil and platelet mediated inflammatory damage to the lung endothelium [22]. Although in malaria the immunopathogenesis is likely also important, in addition sequestration of parasitised red cells may damage the endothelial lining of the lung vasculature, leading to increased vascular permeability and increased extravascular lung water. Pulmonary oedema often develops after start of therapy [23–25]. In this study, patients with severe malaria did not develop pulmonary oedema, which could be partly explained by the restricted fluid management practiced for this patient group. Development of pulmonary oedema was much more common (36% of patients) in earlier studies in the same hospital using aggressive fluid replacement in severe malaria [26].

This study has a number of limitations. First, this was a single centre study in a tertiary care hospital in Bangladesh. However, the study setting was representative of hospitals in the region and to other low–income countries. The number of patients with severe malaria was relatively small in this study, but the complete series of sequential LUS examinations did show the ability to detect the development of new complications other than ARDS over time. Second, the study was conducted in the absence of a reference standard for imaging of pulmonary abnormalities. It was not possible to perform CT scans for patients enrolled in the study. We found a low positive agreement of 56% and a fair negative agreement 90% between LUS and chest radiographs. The lower positive agreement was probably due to poor quality of available bedside chest radiographs and a higher number of positive findings by LUS. During the time of the study we did not have access to reliable blood culture facilities as these were unavailable, which limited the assessment of causes of sepsis. This reflects diagnostic challenges at this study site, similar to many resource-limited healthcare settings in low-income countries.

There are major advantages to having LUS available in a resource-constrained hospital. Using our standard operating procedure and with a battery powered portable ultrasound machine, we were able to perform a chest scan in less than fifteen minutes at the bedside and with high patient tolerability [27]. In the absence of other diagnostic imaging facilities, or where the quality of available CXR is poor, the availability of an ultrasound machine can expedite the underlying diagnosis of severe respiratory distress. Additional studies are warranted to evaluate the role for LUS in guiding fluid management in critically ill patients.

In conclusion, we describe lung aeration patterns in patients with malaria or sepsis by lung ultrasound and demonstrate the important potential benefit of point-of-care LUS in diagnosing pulmonary complications. LUS was highly feasible and allowed to accurately identify patients at risk of death in a resource limited setting.

## Supporting information

S1 Dataset Baseline characteristics and lung ultrasound findings. (CSV)

S2 Dataset Longitudinal lung ultrasound findings. (CSV)

S1 Fig Distribution of lung regions across the chest containing B-patterns measured at baseline in survivors and non-survivors. (TIFF)

S2 Fig Distribution of lung regions across the chest containing B-patterns measured at baseline in patients with and without ARDS. (TIFF)
